# The Effect of* Xialiqi* Capsule on Testosterone-Induced Benign Prostatic Hyperplasia in Rats

**DOI:** 10.1155/2018/5367814

**Published:** 2018-09-30

**Authors:** Hongcai Cai, Guowei Zhang, Zechen Yan, Xuejun Shang

**Affiliations:** ^1^Department of Andrology, Jinling Hospital Affiliated to Southern Medical University, Nanjing, Jiangsu 210002, China; ^2^Family Planning Research Institute/Center of Reproductive Medicine, Tongji Medical College, Huazhong University of Science and Technology, Wuhan, Hubei 430030, China; ^3^Department of Surgery, The First Affiliated Hospital, Zhengzhou University, Zhengzhou, Henan 450052, China

## Abstract

Benign prostatic hyperplasia (BPH) is common among elderly men, of which inflammation, oxidative stress, proliferative, and apoptotic changes play important roles.* Xialiqi* (*XLQ*) capsule, a traditional Chinese herbal formula, is used as a potential drug in treating BPH. This study aims to evaluate the therapeutic effect of* XLQ* capsule on testosterone propionate- (TP-) induced BPH in rats. Fifty male Sprague-Dawley rats were randomly divided into 5 groups: sham control, BPH model, high and low dose of* XLQ*, and finasteride as a positive control group. All groups were treated with appropriate drugs/normal saline for 28 consecutive days. Prostate weights were recorded; histopathological changes and content of IL-8, TNF-*α*, DHT, SOD, MDA, caspase-3, and PCNA of the prostate were determined. Animals with BPH demonstrated significantly increased prostate weights and prostate index, higher levels of IL-8, TNF-*α*, DHT, MDA, and PCNA, but lower activity of SOD and reduced expression of caspase-3. After treatment with* XLQ*, significant reductions of prostate weights, prostate index, IL-8, TNF-*α*, DHT, MDA, and PCNA, increased activity of SOD, and higher level of caspase-3 were shown. The present study indicates that* XLQ* can effectively prevent the development of TP-induced BPH model through mechanisms of anti-inflammation, antioxidation, antiproliferation, and proapoptosis.

## 1. Introduction

Benign prostatic hyperplasia (BPH) is a common andrological disease among the elderly males. BPH is remarkably characterized by histological proliferation of the epithelial cells in the transitional zone of the prostate which leads to lower urinary tract symptoms (LUTS). The constriction of the urethra can result in increased frequency, urgency and hesitancy of urination, and compromised urine flow, which eventually impacts the quality of life [1, 2]. Up to now, the pathophysiology of BPH is not fully clarified, although many attempts have been made in the past decades.

Commonly, testicular hormones and aging are two core elements attributed to the genesis and development of BPH. Moreover, pathways of proliferation/apoptosis and inflammation as long as oxidation/antioxidation are considered to be involved in the development of BPH, which have been supported by many researches during the past decades [3–7].

Medicinal therapy remains the first line treatment for most patients. Given the importance of DHT in the development of BPH, inhibitors of 5*α*-reductase (e.g., finasteride and dutasteride) which prevents the conversion of DHT from testosterone and reduces DHT level and thereby suppresses hyperplastic growth of the prostate are used in the clinical treatment of BPH [8]. Nevertheless, finasteride-associated untoward reactions are regularly reported, including gynecomastia, headache, dizziness, chest pain, upper respiratory infections, decrease libido, erectile dysfunction, and male infertility due to a reduced sperm count [9–12]. Such side effects cause the limitation of conventional drugs used for BPH and, nevertheless, might be prevented by other safe agents.

*Xialiqi* (*XLQ*) capsule is a traditional Chinese herbal formula, considered as a multicomponent agent, composing of eight herbs (*Astragali Radix*,* Ligustri Lucidi Fructus*,* Cinnamoni Cortex*,* Talcum*,* Phellodendri Amurensis Cortex*,* Amber*,* Prunellae Spica*, and* Litchi Semen*, see in Table 1), which as a whole produces an effect of antiproliferation, proapoptosis, anti-inflammation, analgesia, and microcirculation improvement as previously reported [13–18]. For instance,* Prunellae Spica*, as one of the main components of* XLQ* capsules, is reported to reduce the levels of the proproliferative factors (Cyclin D1 and CDK4), as well as downregulate the proangiogenic factors and increase the proapoptotic Bax/Bcl-2 rate [17]. And* Litchi Semen* can inhibit the proliferation of HepG2 cells, which may be concerned with the property of proapoptosis [15]. Another major component,* Astragali Radix*, is reported to play a potential anti-inflammatory activity, which is widely used in oriental medicine for tonifying the immune response and improving circulation [14]. Recently,* XLQ* capsule has been used in the treatment of BPH at the early and midstage, achieving a sound result as those clinical drugs (unpublished). Although previous studies have observed the pharmacological effects of similar traditional herbal formula [4, 19–22], there have been no studies of* XLQ* capsule on its possible protective effects against BPH.

Therefore, the current study was aiming to investigate the role of* XLQ* capsule in the development of BPH by establishing a testosterone-induced BPH model in rats. Our research will help to provide an experimental basis for the development of new agents for the prevention and treatment of BPH on clinic.

## 2. Material and Methods

### 2.1. Drugs

The herbal medicine of* XLQ* capsule was purchased from Shijiazhuang Yiling Pharmaceutical Co., Ltd. (Shijiazhuang, Hebei, China, Z20123085) in February 2015. The composition of* XLQ* capsule is listed in Table 1. The powder of the medicine was dissolved in adequate normal saline (NS) according to different doses and stored in 4°C before use.

### 2.2. Animals

Fifty specific pathogen free (SPF) grade adult male Sprague-Dawley (S-D) rats weighed 200 to 250 g were purchased from Department of Comparative Medicine, Jinling Hospital Affiliated to Nanjing University School of Medicine (Nanjing, Jiangsu, China) for the study. The rats were kept on a standard laboratory diet and water* ad libitum* and were adapted to laboratory environment (20-26°C) under a 12 h light-dark cycle for one week before experiment. All experiments were performed according to the National Institutes of Health Guide for the care and use of Laboratory Animals and international ethical guidelines and were approved by Institutional Animal Care and Use Committee of Jinling Hospital Affiliated to Nanjing University School of Medicine (SYXK (M) 2012-0047).

### 2.3. Establishment of Testosterone Propionate-Induced Rat Model of BPH

Establishment of BPH rat model and experimental procedures were carried out as previously described [7]. The rats were assigned into five groups randomly (10 rats/group). And with the exception of the sham operation control group, four groups of rats were anesthetized with intraperitoneal injection of phenobarbital (50 mg/kg) and castrated aseptically to remove bilateral testes. All castrated rats were injected with testosterone propionate (TP, Zhejiang Xianju Pharmaceutical Co., Ltd., Taizhou, Zhejiang, China) 0.5 mg/kg/d (dissolved in corn oil), subcutaneously (SC), one week after castration. The BPH model was induced through 28 consecutive days of TP treatment, which was further confirmed by histopathological examination of prostate tissue. Then one group was served as a model group while the other three served as experimental groups. The experimental groups were treated with high dose (1.20 g/kg/d) and low dose (0.61 g/kg/d) of* XLQ* by intragastric administration (i.g.) for 28 days. Finasteride dissolved in ultrapure water (0.8 mg/kg/d, i.g., Hangzhou Merck, China; J20120071) was served as a positive control group.

Body weight (BW) of the rats were recorded every week throughout the whole study. Following the final injection and overnight fasting, the rats were anesthetized with pentobarbital (50 mg/kg, intraperitoneal injection, i.p.). Blood samples were collected, and prostate tissues were immediately separated and weighed. Prostate lobes sections were fixed with 4% paraformaldehyde for histological and immunohistochemical analysis, and the remaining prostate sections were stored at -80°C for further analysis.

### 2.4. Assessment of Prostate Weight (PW) and Prostatic Index (PI)

PW and BW of the rats were recorded in all groups. The PI was computed as PW/BW ×100%, and the mean PI ratios were computed in each group. The percentage of inhibition of PW and PI was computed as follows: 100–[(T–C)/(B–C) ×100], where C, B, and T are the values of the control group, BPH group, and treatment group, respectively.

### 2.5. Histopathological Examination

The prostate tissues were fixed in 10% formalin immediately for 24 h. Prostate samples were then paraffin-embedded and sectioned at thickness of 5 *μ*m. After dewaxing and rehydration, prostate sections were mounted on slides and stained with hematoxylin and eosin (H&E) for routine histological examination under a light microscopy (Olympus, Tokyo, Japan).

### 2.6. ELISA Assay of Interleukin- (IL-) 8, Tumor Necrosis Factor (TNF)-*α*, and DHT

Expressions of IL-8, TNF-*α*, and DHT in the serum and prostate tissues were measured by enzyme-linked immunosorbent assay (ELISA) kits according to the manufacturer's instructions (AMEKO Inc., Shanghai, China). The absorbance was recorded at 450 nm and values are recorded as per mL for serum and per mg protein for the prostate.

### 2.7. Immunohistochemical Staining of Proliferating Cell Nuclear Antigen (PCNA)

Immunohistochemistry was carried out on thickness of 4 *μ*m sections after deparaffinization. Antigen was retrieved by citrate buffer (pH 6.0) with 95°C (microwave) for 10 min, followed by peroxides quenching with 3 % H_2_O_2_ in PBS for 10 min. After washing with PBS, the sections were preblocked with normal goat serum for 10 min. The slides were incubated with the primary antibody, anti-PCNA (mouse monoclonal antibody; ab29, Abcam, Cambridge, UK), in a dilution of 1: 200 for overnight at 4°C. Next, the sections were incubated with biotinylated secondary antibodies, in a dilution of 1: 1000, for 1 h. Following a washing step with PBS, the streptavidin-HRP was added. Lastly, the sections were rinsed with PBS and developed with diaminobenzidine tetrahydrochloride substrate (DAB) in 10 min. Each of the sections was observed for more than three random regions at × 200 and × 400.

### 2.8. Measurement of Superoxide Dismutase (SOD) Activity and Malondialdehyde (MDA) Concentration

SOD activity in the serum and prostate were determined by a commercially available kit (Beyotime, Nantong, China) following the manufacturing instructions. The absorbance was determined at 450 nm, and SOD activity is expressed as U/L serum and U/mg protein. Concentration of MDA in the serum and prostate were detected by a commercial kit (AMEKO Inc., Shanghai, China) following the manufacturer's instructions. The absorbance was also determined at 450 nm after adding stop solution within 10min, and MDA values are expressed as nmol MDA/L serum pmol MDA/g protein.

### 2.9. Immunofluorescent Assay of Caspase-3

Similar experimental procedures were carried out as those in immunohistochemistry until the step of primary antibody reaction, of which the slides were incubated, with anti-caspase-3 (rabbit monoclonal antibody; ab32150, Abcam, Cambridge, UK) in a dilution of 1:100 for overnight at 4°C. After three times of rinsing with PBS, sections were incubated with secondary antibody, at a dilution of 1:500 in PBS with 1.5% normal blocking serum at room temperature for 50 min. The slides were rinsed three times with PBS and were counterstained with DAPI (sc-3598, Santa Cruz Biotech, CA, USA) to visualize the nuclei.

### 2.10. Statistical Analysis

Graph Pad Prism 6.0 (GraphPad Software, Inc., La Jolla, CA, USA) was adopted for statistical analyses. Data are expressed as the mean ± SEM. One-way ANOVA was used for comparisons of group data followed by the post hoc test (Dunnett's test). The 0.05 level of probability was considered as statistical significant.

## 3. Results

### 3.1. *XLQ* Decreased the PW and PI of BPH Rats

**Figure 1:**
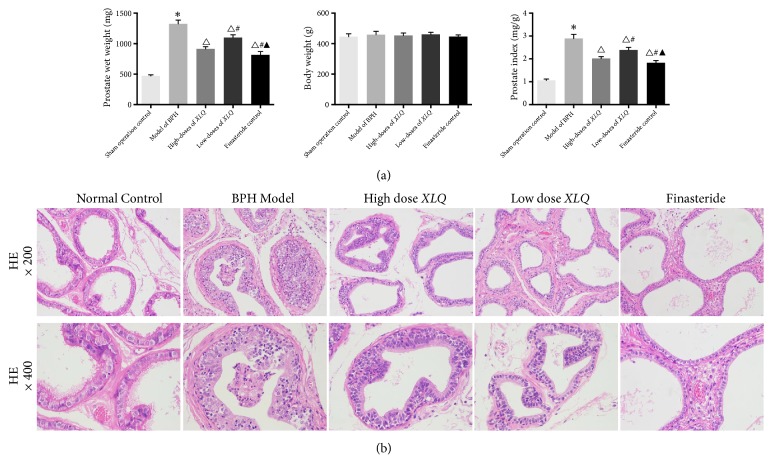
Effects of* XLQ* on the histopathological appearance of the prostate, prostate weight, body weight, and prostate index of BPH rats. (a) Effects of* XLQ* on prostate weight, body weight, and prostate index. (b) Effects of* XLQ* on the histopathological appearance of the prostate. Up: hematoxylin and eosin (H&E) staining ×200; down: H&E staining ×400. *∗*:* versus *the sham operation control group,* P<*0.01; Δ:* versus *the model of BPH group,* P<*0.01; #:* versus *the high-dose* XLQ* group,* P<*0.01; ▲:* versus *the low-dose* XLQ* group,* P<*0.01. Error bars indicate SEM.

Rats in the BPH group demonstrated significantly greater PW and PI than those in the sham operation control group, while PW in the* XLQ*-treated groups were markedly reduced compared to the model group (Table 2 and Figure 1(a)). Finasteride-treated group also exhibited with obvious decreases in PW and PI. No significant differences in BW changes were observed among groups. Compared with the control, the percentages of inhibition on PW in high dose of* XLQ*, low dose of* XLQ*, and finasteride groups were 48.18%, 26.49%, and 59.54%, respectively, and the percentages of inhibition on PI were 47.54%, 27.32%, and 57.92%, respectively (Table 2).

### 3.2. *XLQ* Prevented the Morphological Changes of Prostatic Tissues in BPH Rats

There were no morphological changes in the lining epithelium or the acini of the sham operation control group. Cuboidal epithelial cells of regular size were observed. However, after TP-induction, disrupted morphology in the prostate epithelia was shown by significant thickening, hypertrophy, and hyperplasia with papillary projections in the lining epithelium or the acini. And widening of the lumen diameter without remarkable expansion in the stroma was also found in the model group.

After treatment, mild epithelial hyperplasia was shown in finasteride-treated group compared with the model group. Similar reduction in epithelial hyperplasia was also shown in* XIQ*-treated animals compared with BPH animals. No remarkable inflammatory cells were detected in the* XLQ*- or finasteride-treated groups (Figure 1(b)).

### 3.3. *XLQ* Alleviated the Inflammation in BPH Rats

**Figure 2:**
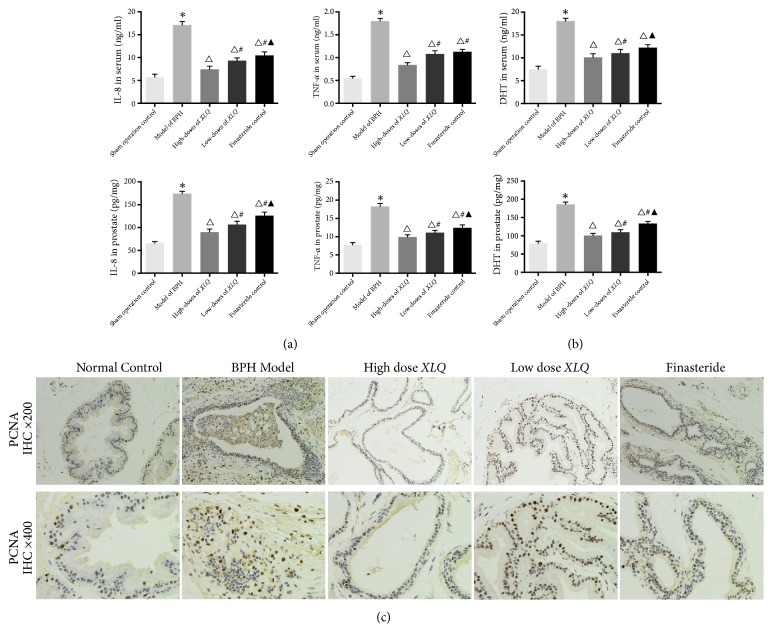
Effects of* XLQ* on the expression levels of IL-8, TNF-*α*, DHT, and PCNA in BPH rats. (a) Effects of* XLQ* on the expression levels of IL-8, TNF-*α* in the serum, and prostate of BPH rats. (b) Effects of* XLQ* on the expression levels of DHT in the serum and prostate of BPH rats. (c) Effects of* XLQ* on the expression of PCNA in the prostate of BPH rats. IHC: immunohistochemistry. *∗*:* versus *the sham operation control group,* P<*0.01; Δ:* versus *the model of BPH group,* P<*0.01; #:* versus *the high-dose* XLQ* group,* P<*0.01; ▲:* versus *the low-dose* XLQ* group,* P<*0.01. Error bars indicate SEM.

A significant increase in serum IL-8 and TNF-*α* level was shown in the BPH group (17.12 ± 0.73 ng/ml and 1.80 ± 0.06 ng/ml, respectively,* P<*0.01) compared with the sham operation control group (5.71 ± 0.65 ng/ml and 0.55 ± 0.04 ng/ml; Figure 2(a)). On the contrary, rats in the finasteride-treated group demonstrated a significantly reduction of serum IL-8 and TNF-*α* level (10.48 ± 0.76 ng/ml and 1.13 ± 0.05 ng/ml, respectively,* P<*0.01) compared with the BPH group. Similar to finasteride-treated group, significant reductions in IL-8 and TNF-*α* levels were also shown in the* XLQ*-treated groups compared with the BPH group (7.42 ± 0.71 ng/ml and 0.84 ± 0.05 ng/ml in the high-dose group, respectively,* P<*0.01; 9.32 ± 0.60 ng/ml and 1.08 ± 0.07 ng/ml in the low-dose group, respectively,* P<*0.01). Moreover, among the treatment groups, high-dose* XLQ* group decreased the most significantly.

In the prostate, similar results of IL-8 and TNF-*α* level were shown, of which the high-dose* XLQ*-treated group (89.86 ± 6.65 ng/mg and 9.91 ± 0.63 pg/mg, respectively,* P<*0.01; Figure 2(a)) decreased the most significantly, compared to the model of BPH group (174.04 ± 5.24 ng/mg and 18.26 ± 0.81 pg/mg, respectively) and finasteride-treated group (126.06 ± 7.52 ng/mg and 12.41 ± 0.81 pg/mg, respectively,* P<*0.01).

### 3.4. *XLQ* Downregulated the Expression of DHT in BPH Rats

A significant increase in serum DHT level was shown in the BPH group compared with the sham operation control group (18.04 ± 0.62 ng/m versus l7.51 ± 0.67 ng/ml,* P<*0.01; Figure 2(b)). On the contrary, a significantly reduction of serum DHT level was displayed in the finasteride-treated group (10.11 ± 0.78 ng/ml,* P<*0.01) compared with the BPH group. Likewise, significant reductions in DHT levels were observed in the* XLQ*-treated groups (11.02 ± 0.80 ng/ml in the high-dose group,* P<*0.01; 12.24 ± 0.66 ng/ml in the low-dose group,* P<*0.01) compared with the BPH group. However, no significant difference was found between the high-dose* XLQ* and finasteride-treated group (*P>*0.05).

In the prostate, similar results of DHT level were shown, of which the finasteride-treated group (100.63 ± 5.78 pg/mg; Figure 2(b)) decreased most significantly, compared to BPH group (186.14 ± 6.26 pg/mg,* P<*0.01) and* XIQ*-treated groups (109.83 ± 6.93 pg/mg in the high-dose group,* P<*0.01; 133.59 ± 5.74 pg/mg in the low-dose group,* P<*0.01).

### 3.5. *XLQ* Inhibited the Expression of PCNA in the Prostate

The expression of PCNA protein was elevated in the prostate tissues among BPH group comparing with the sham group. After treatment, the expression of PCNA reduced in the finasteride-treated group compared to the BPH group. Reduction of expression of PCNA protein was also observed in the* XIQ*-treated groups in comparison to the BPH group (Figure 2(c)).

### 3.6. *XLQ* Regulated the Level of SOD and MDA in BPH Rats

**Figure 3:**
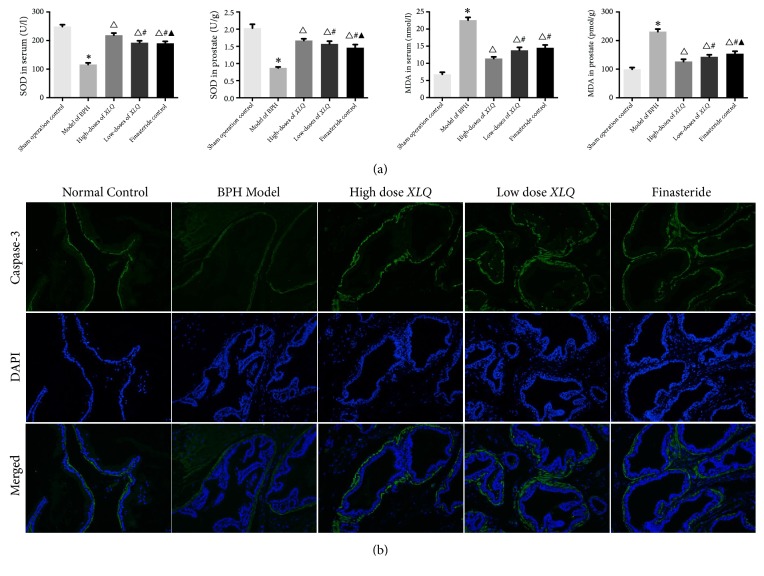
Effects of* XLQ* on the activity of SOD, concentration of MDA, and expression level of caspase-3 in BPH rats. (a) Effects of* XLQ* on the activity of SOD and concentration of MDA in the serum and prostate of BPH rats. (b) Effects of* XLQ* on the expression of caspase-3 in the prostate of BPH rats. Magnification: ×200. *∗*:* versus *the sham operation control group,* P<*0.01; Δ:* versus *the model of BPH group,* P<*0.01; #:* versus *the high-dose* XLQ* group,* P<*0.01; ▲:* versus *the low-dose* XLQ* group,* P<*0.01. Error bars indicate SEM.

A significant decreased activity in serum SOD level was discovered in the BPH group compared with the sham operation control group (116.05 ± 6.06 U/L versus 248.80 ± 6.49 U/L,* P<*0.01; Figure 3(a)). Conversely, a significantly increase activity in serum SOD level was revealed in the finasteride-treated group (190.34 ± 6.66 U/L,* P<*0.01) compared to the model group. Likewise, the* XLQ*-treated groups also demonstrated significant higher SOD levels in serum (218.52 ± 7.83 U/L and 192.72 ± 6.38 U/L in the high- and low-dose group, respectively,* P<*0.01). Moreover, among the treatment groups, high-dose* XLQ* group increased the most significantly, but no significant difference was exhibited between the low-dose* XLQ* and finasteride-treated group (*P>*0.05). Conversely, significant increased content of MDA in serum was indicated in the model of BPH group (22.62 ± 0.78 nmol/L,* P<*0.01), compared to sham operation control group (6.82 ± 0.60 nmol/L). Among the treatment groups, high-dose* XLQ* group seemed to decrease the most significantly (11.40 ± 0.51 nmol/L). And no difference was observed between the low-dose* XLQ* and finasteride-treated group.

In the prostate, similar results of SOD and MDA level were shown, of which the high-dose* XLQ*-treated group (1.67 ± 0.05 U/g and 126.81 ± 7.84 pmol/g, respectively,* P<*0.01; Figure 3(a)) changed the most significantly, compared to BPH group (0.87 ± 0.03 U/g and 231.08 ± 9.40 pmol/g, respectively,* P<*0.01) and finasteride-treated group (1.46 ± 0.09 U/g and 154.39 ± 8.53 pmol/g, respectively,* P<*0.05).

### 3.7. *XLQ* Promoted the Expression of Caspase-3 in the Prostate

The expression of caspase-3 protein significantly decreased in the prostate tissues among BPH group compared to the sham group. Expression of caspase-3 protein was reduced in the* XIQ*-treated groups comparing to the BPH group. Furthermore, an increase of the expression of caspase-3 was also observed in the finasteride-treated group after treatment (Figure 3(b)).

## 4. Discussion

In the current study, we discovered that* XLQ* capsule (1.20 g/kg/d and 0.61 g/kg/d, i.g. for 28 d) treatment could significantly inhibit the development of TP-induced BPH, which was confirmed by reduction in elevated PW, PI, and histopathological changes. Compared with the BPH rats, reductions in IL-8, TNF-*α*, DHT, MDA, and PCNA levels in both the prostate and serum and increases in SOD and caspase-3 were found, suggesting that* XLQ* might be an effective drug for BPH treatment. Above all, this study will be the first to provide experimental evidence for the wider application of* XLQ* capsule in clinics in the future.

In previous studies using rat models, changes in PW and histomorphology have been an important indicator for the inhibitory effects of substances on the development of BPH [23, 24]. BPH is commonly characterized by hyperplasia of the epithelium and stroma in the prostate, which results in an increase of the PW. The hyperplastic prostate tissue may gradually constrict the urethral canal to cause partial or sometimes even complete obstruction, leading to lower urinary tract obstruction [25]. For these reasons, by monitoring the changes of PW, previous studies have tested the inhibitory roles of various agents on BPH development. In the present study,* XLQ* reduced the PW, PI, and histological abnormalities in TP-induced BPH rats, consistently as previous studies [23, 24], which supports the idea that* XLQ* inhibits BPH development. What is more,* XLQ* at a dose of 1.20 g/kg/d seemed to achieve maximum efficacy in this BPH rat model.

Inflammation is commonly presented in BPH, which may cause tissue injury; and cytokines, secreted from inflammatory cells, can drive angiogenesis and local growth factor production in the tissues as a self-protection response [26]. IL-8 and TNF-*α*, as proinflammatory cytokines, which are considered as potent growth factors for prostatic epithelial and stromal cells, increase in BPH models according to previous studies [7, 27]. Thus, agents with the properties of anti-inflammation in BPH have been reported by many researches [28, 29]. In our study, significant reduction of TNF-*α* and IL-8 levels was determined in both serum and prostate in* XLQ*-treated groups, compared to control groups. This might suggest that anti-inflammation is involved in the mechanisms of* XLQ* capsule in treating BPH, although more investigation of other cytokines would further strengthen our conclusion.

DHT, an active metabolite of testosterone catalyzed by 5*α*-reductase, is an important causative factor in BPH development [30]. DHT can easily bind to ARs due to its higher affinity to the androgen receptor (AR), which stimulates the growth for the epithelial and stromal cells in the prostate [8]. Therefore, DHT is basically responsible for prostatic epithelial and stromal cell hyperplasia [31]. Finasteride, as an elective drug targeting on 5*α*-reductase, has been reported to reduce DHT levels in the serum and prostate of BPH [32, 33]. However, because of the adverse effects, the use of 5*α*- reductase inhibitors (e.g., dutasteride and finasteride) has been limited [12, 34]. In our current study, significant reductions in DHT levels of the serum and prostate were observed in* XLQ*-treated groups compared with the BPH model group, as did the finasteride-treated group.

Oxidative stress can possibly cause damage to the cells, tissues, even to organs by impairing important biomolecules and cells, which is considered as an important factor accounting for the pathogenesis of BPH [35, 36]. Conversely, antioxidants, usually exist as compounds or enzymes, could compete with oxidative substrates, thus protecting the cellular structure [37]. Previous studies have demonstrated reductions of antioxidant levels in both serum and prostate of BPH animals, while increasing oxidant in BPH model [22, 24]. In our study, we have found increased activity of SOD and decreased content of MDA in* XLQ*-treated groups, compared to control groups, of which high dose of* XLQ* group changed the most significantly, which is similar to previous studies. These findings support the opinion that* XLQ *capsule may treat BPH through the mechanism of antioxidation.

PCNA, a 36-kD DNA polymerase delta auxiliary protein, is specifically expressed in proliferating cell nuclei. By far, the expression of PCNA in cell has been identified as a marker for the G1/S phase of the cell cycle, which is related to the pathogenesis of BPH development [29, 38]. In our present study, a significant increased PCNA expression was detected in the model group. However, this could be inhibited by* XLQ* administration, as evidenced by immunohistochemistry. Caspase-3, an apoptotic biomarker, is crucial for apoptosis involved in the pathogenesis of BPH as testified by many studies [3, 28]. Consistent with previous reports, our study showed that* XLQ* administration significantly increased the caspase-3 levels in a TP-induced BPH rat model. These findings might suggest that the effects of* XLQ* on BPH development involve antiproliferative and proapoptotic activity although more researches are still needed.

The drawback inherent to this study is that we did not carry out experiments about the side effects of* XLQ* capsule, such as hepatotoxicity and nephrotoxicity. Therefore, it is necessary to monitor liver and renal function during* XLQ* treatment in the future study. In addition, due to castrated model of BPH, it is impossible to detect the influence on male reproductive system along with the long-term treatment. It is still unknown which ingredient of the compounds conduces to the activity of anti-BPH. Moreover, the specific signaling pathways involved to play the role of bioactivity remains elusive. Last, rodent model of BPH used in this study is indeed rather different from human beings [39], which will limit the application of our results for human beings. Accordingly, more researches upon the cellular and molecular levels, like the involvement of androgen and androgen receptor pathways, are still needed to further elucidate the underlying mechanisms of* XLQ* in the treatment of BPH.

In conclusion, this study firstly demonstrates that* XLQ* capsule can reduced PW and PI and protect the morphology of prostate tissue by the mechanisms of anti-inflammation, antioxidation, downregulation of DHT, antiproliferation, and proapoptosis of experimental BPH rats. Therefore, these results reveal that* XLQ* capsule effectively inhibits the development of TP-induced BPH rat model, which strongly supports the application of using* XLQ* capsule therapeutically in the treatment of BPH in future.

## Figures and Tables

**Table 1:** Composition of *XLQ* capsule.

**Latin name**	**Amount (g)**	**Ratio (**%**)**	**Source**
*Astragali Radix*	70	46.1	Inner Mongolia, China

*Ligustri Lucidi Fructus*	14	9.2	Zhejiang, China

*Cinnamoni Cortex*	2.8	1.8	Guangxi, China

*Talcum*	14	9.2	Liaoning, China

*Phellodendri Amurensis Cortex*	7	4.6	Jilin, China

*Amber*	2.1	1.4	Liaoning, China

*Prunellae Spica*	21	13.8	Jiangsu, China

*Litchi Semen*	21	13.8	Guangdong, China

**Total**	**151.9**	**100**	

**Table 2:** Effects of *XLQ* capsule on prostate weight, body weight, and prostate index.

**Group ** **(n=10)**	**PW (mg)**	**BW (g)**	**PI (mg/g)**	%**Inhibition**	
				**PW**	**PI**
Sham operation control	471.38±17.96	445.56±19.27	1.06±0.06	/	/

BPH model control	1326.15±60.20^*∗*^	459.44±21.10	2.89±0.18^*∗*^	/	/

High-dose *XLQ *Capsule	914.33±36.08^Δ^	453.44±16.08	2.02±0.08^Δ^	48.18	47.54

Low-dose *XLQ* Capsule	1099.76±46.28^Δ#^	460.56±13.48	2.39±0.11^Δ#^	26.49	27.32

Finasteride	817.25±53.99^Δ#▲^	446.00±11.08	1.83±0.10^Δ#▲^	59.54	57.92

*∗*: *versus *the sham operation control group, *P*<0.01; Δ: *versus *the model of BPH group, *P*<0.01; #: *versus *the high-dose *XLQ* group, *P*<0.01; ▲: *versus *the low-dose *XLQ* group, *P*<0.01; BW: body weight; PI: prostate index; PW: prostate weight; *XLQ*: *Xialiqi*.

## Data Availability

All data generated or analyzed during this study are included in this article (and its supplementary materials).

## Supplementary Materials

Supplementary Materials
** Supplementary Table S1: **effects of* XLQ* on the level of IL-8 and TNF-*α* in the serum and prostate.** Supplementary Table S2: **effects of* XLQ* on the concentration of DHT in the serum and prostate.** Supplementary Table S3: **effects of* XLQ* on the activity of SOD and concentration of MDA in the serum and prostate.
