# Supplementary material for: The Effect of Xialiqi Capsule on Testosterone-Induced Benign Prostatic Hyperplasia in Rats
Source: Evid Based Complement Alternat Med. 2018 Sep 30;2018:5367814. doi: 10.1155/2018/5367814 (PMC6186362; doi:10.1155/2018/5367814)
Supplement: Supplementary Materials — Supplementary Table S1: effects of XLQ on the level of IL-8 and TNF-α in the serum and prostate. Supplementary Table S2: effects of XLQ on the concentration of DHT in the serum and prostate. Supplementary Table S3: effects of XLQ on the activity of SOD and concentration of MDA in the serum and prostate. [file 5367814.f1.docx]

**The effect of *Xialiqi* capsule on testosterone-induced benign prostatic hyperplasia in rats**

**Hongcai Cai, ^1, 2^ Guowei. Zhang, ^1^ Zechen Yan, ^3, *^ Xuejun Shang^1, *^**

*^1^ Department of Andrology, Jinling Hospital Affiliated to Southern Medical University,* *Nanjing, Jiangsu 210002, China*

*^2^ Family Planning Research Institute/Center of Reproductive Medicine, Tongji Medical College, Huazhong University of Science and Technology, Wuhan, Hubei 430030, China*

*^3^ Department of Surgery, the First Affiliated Hospital, Zhengzhou University, Zhengzhou, Henan 450052, China*

**Corresponding Author’s information**: Xuejun Shang, M.D., Ph.D. Department of Andrology, Jinling Hospital Affiliated to Southern Medical University, Nanjing, Jiangsu 210002, China. Tel: +86-13813905418; Fax: +86-025-80863168; E-mail: shangxj98@163.com

Zechen Yan, M.D., Ph.D. Department of Surgery, the First Affiliated Hospital, Zhengzhou University, Zhengzhou, Henan 450052, China. Tel: +86-13526782266; E-mail: yanzechen@qq.com

^*^Contributed equally

**Supplementary data**

**Supplementary Table S1** Effects of *XLQ* on the level of IL-8 and TNF-α in the serum and prostate.

**Supplementary Table S2** Effects of *XLQ* on the concentration of DHT in the serum and prostate.

**Supplementary Table S3** Effects of *XLQ* on the activity of SOD and concentration of MDA in the serum and prostate.

**Supplementary Table S1** Effects of *XLQ* on the level of IL-8 and TNF-α in the serum and prostate.

| **Group (N=10)** | **IL-8 in serum (ng/ml)** | **IL-8 in prostate (ng/mg)** | **TNF-α in serum (ng/ml)** | **TNF-α in prostate (pg/mg)** |
| --- | --- | --- | --- | --- |
| Sham operation control | 5.71±0.65 | 65.81±3.52 | 0.55±0.04 | 7.74±0.64 |
| Model of BPH | 17.12±0.73^*^ | 174.04±5.24^*^ | 1.80±0.06^*^ | 18.26±0.81^*^ |
| High-dose *XLQ* | 7.42±0.71^Δ^ | 89.86±6.65^Δ^ | 0.84±0.05^Δ^ | 9.91±0.63^Δ^ |
| Low-dose *XLQ* | 9.32±0.60^Δ#^ | 106.45±7.06^Δ#^ | 1.08±0.07^Δ#^ | 11.12±0.60^Δ#^ |
| Finasteride | 10.48±0.76^Δ#▲^ | 126.06±7.52^Δ#▲^ | 1.13±0.05^Δ#^ | 12.41±0.81^Δ#▲^ |

*: *versus* the sham operation control group, *P <* 0.01; Δ: *versus* the model of BPH group, *P <* 0.01; #: *versus* the high-dose *XLQ* group, *P <* 0.01; ▲: *versus* the low-dose *XLQ* group, *P <* 0.01; BW: body weight; PI: prostate index; PW: prostate weight; *XLQ*: *Xialiqi*.

**Supplementary Table S2** Effects of *XLQ* on the concentration of DHT in the serum and prostate.

| **Group (N=10)** | **DHT in serum (ng/ml)** | **DHT in prostate (pg/mg)** |
| --- | --- | --- |
| Sham operation control | 7.51±0.67 | 79.32±6.06 |
| Model of BPH | 18.04±0.62^*^ | 186.14±6.26^*^ |
| High-dose *XLQ* | 11.02±0.80^Δ^ | 109.83±6.93^Δ^ |
| Low-dose *XLQ* | 12.24±0.66^Δ#^ | 133.59±5.74^Δ#^ |
| Finasteride | 10.11±0.78^Δ&^ | 100.63±5.78^Δ#&^ |

*: *versus* the sham operation control group, *P <* 0.01; Δ: *versus* the model of BPH group, *P <* 0.01; #: *versus* the high-dose *XLQ* group, *P <* 0.01; ▲: *versus* the low-dose *XLQ* group, *P <* 0.01.

**Supplementary Table S3** Effects of *XLQ* on the activity of SOD and concentration of MDA in the serum and prostate.

| **Group**  **(n=10)** | **SOD in serum (U/L)** | **SOD in prostate (U/g)** | **MDA in serum (nmol/L)** | **MDA in prostate (pmol/g)** |
| --- | --- | --- | --- | --- |
| Sham operation control | 248.80±6.49 | 2.03±0.11 | 6.82±0.60 | 99.73±5.97 |
| Model of BPH | 116.05±6.06^*^ | 0.87±0.03^*^ | 22.62±0.78^*^ | 231.08±9.40^*^ |
| High-dose *XLQ* | 218.52±7.83^Δ^ | 1.67±0.05^Δ^ | 11.40±0.51^Δ^ | 126.81±7.84^Δ^ |
| Low-dose *XLQ* | 192.72±6.38^Δ#^ | 1.57±0.08^Δ#^ | 13.82±0.83^Δ#^ | 143.49±7.03^Δ#^ |
| Finasteride control | 190.34±6.66^Δ#^ | 1.46±0.09^Δ#▲^ | 14.56±0.81^Δ#^ | 154.39±8.53^Δ#▲^ |

*: *versus* the sham operation control group, *P <* 0.01; Δ: *versus* the model of BPH group, *P <* 0.01; #: *versus* the high-dose *XLQ* group, *P <* 0.01; ▲: *versus* the low-dose *XLQ* group, *P <* 0.01.
